# Editorial: Blood-Brain Barrier Dysregulation and Recovery Following Brain Ischemia: Cellular Constituents, Molecular Mechanisms, and Therapeutic Strategies Enabling Successful Brain Remodeling

**DOI:** 10.3389/fncel.2022.968425

**Published:** 2022-07-06

**Authors:** Hansen Chen, Zhijuan Cao, Yong Gu, Dirk M. Hermann

**Affiliations:** ^1^Department of Neurosurgery, Stanford University School of Medicine, Stanford, CA, United States; ^2^Department of Neurosurgery, Stanford Stroke Center, Stanford University School of Medicine, Stanford, CA, United States; ^3^Clinical Research Center, Hainan Provincial Hospital of Traditional Chinese Medicine, Haikou, China; ^4^Department of Neurology, University Hospital Essen, Essen, Germany

**Keywords:** stroke, blood-brain barrier, stem cell, exosome, hemorrhagic transformation (HT), biomarker

Current treatments for ischemic stroke are limited to reperfusion therapy through thrombolysis and thrombectomy (Shi et al., 2018). Despite extensive preclinical findings on methods promoting neuroprotection after stroke, the translations to clinical trials are lost. The failure of translations in acute stroke treatment raises doubts about the current strategy of only targeting neurons. The initial stroke injury disrupts the blood-brain barrier (BBB) and results in a leaky BBB, allowing undesirable circulating factors to flow into the brain parenchyma. Imagine a house that is flooded; it will be difficult to salvage our belongings (neurons) unless the flooding (BBB leakage) is under control. To protect the brain after stroke injury, it is vital to understand the mechanisms of BBB leakage and devise treatments to save the BBB. This special issue focuses on the current breakthroughs in the prediction, mechanisms and treatments of BBB damage in stroke.

Saving the BBB is critical not only for developing new therapies but also for improving thrombectomy or thrombolysis. Thrombectomy and thrombolysis can increase the risk of hemorrhagic transformation (HT) and worsen the stroke outcomes (Spronk et al., 2021). This complication limits the use of these interventions. To address this, identifying methods to predict HT in patients with acute ischemic stroke prior to reperfusion therapy can maximize benefits while minimizing risks. In this special issue, Qi et al. used isobaric tags for relative and absolute quantification (iTRAQ) based proteomic analysis to screen differentially expressed protein in plasma samples from stroke patients. Their findings suggest the potential biomarkers for predicting HT after ischemic stroke. Yuan et al. reported that combining serum occludin and NIHSS score enhanced the accuracy of predicting HT. This finding suggests that a combination of molecular markers and NIHSS scores can contribute to identifying patients who can benefit from reperfusion therapy and minimize their risk of HT.

Understanding the mechanisms of BBB damage will be crucial for developing therapeutics for BBB protection and reducing HT. Janssen et al. showed that fatty acid inhibition worsened ischemic stroke injury *in vivo* and *in vitro*, with increased cell death, enlarged brain infarct, and impeded functional recovery. The breakdown of the BBB caused by fatty acid inhibition is linked to aforementioned worsening outcomes. This study revealed the important role of fatty acids in BBB function during ischemic stroke, providing a potential target for ischemia treatment.

Exosome treatment may modulate the neurovascular unit and provide protection against stroke injury (Zagrean et al., 2018). Huang et al. showed that exosomes produced from healthy rat serum protected rodents from cerebral ischemia-reperfusion injury, as seen by reduced brain infarction and improved neurological/behavioral scores. The therapeutic efficacy could be linked to the suppression of cell apoptosis as well as the protection of the BBB *via* modulating autophagy and preserving tight junction proteins. This pre-clinical study has enormous translational potential, highlighting the use of healthy plasma exosomes/extracellular vehicles for stroke treatment, particularly BBB protection.

Stem cell therapy holds great promise for stroke treatment (Boshuizen and Steinberg, 2018; Li et al., 2021). Yang et al. summarized the recent advances in mesenchymal stem cell (MSC) treatment for hemorrhagic stroke. The protective effects of MSC may result from brain restoration, anti-inflammation, anti-oxidative stress, and autophagy modulation. Trophic factors and extracellular vesicles from MSC were discussed. In terms of clinical translation, safety and better simulation of diseases in pre-clinical models, source of MSC, dosage, route, time point, and mechanisms are suggested for further investigation.

In summary, these studies provide strategies for HT prediction and advance our understanding of BBB protection for stroke treatment (Figure 1). These findings will be useful in translating preclinical findings into therapeutic stroke treatment.

**Figure 1:**
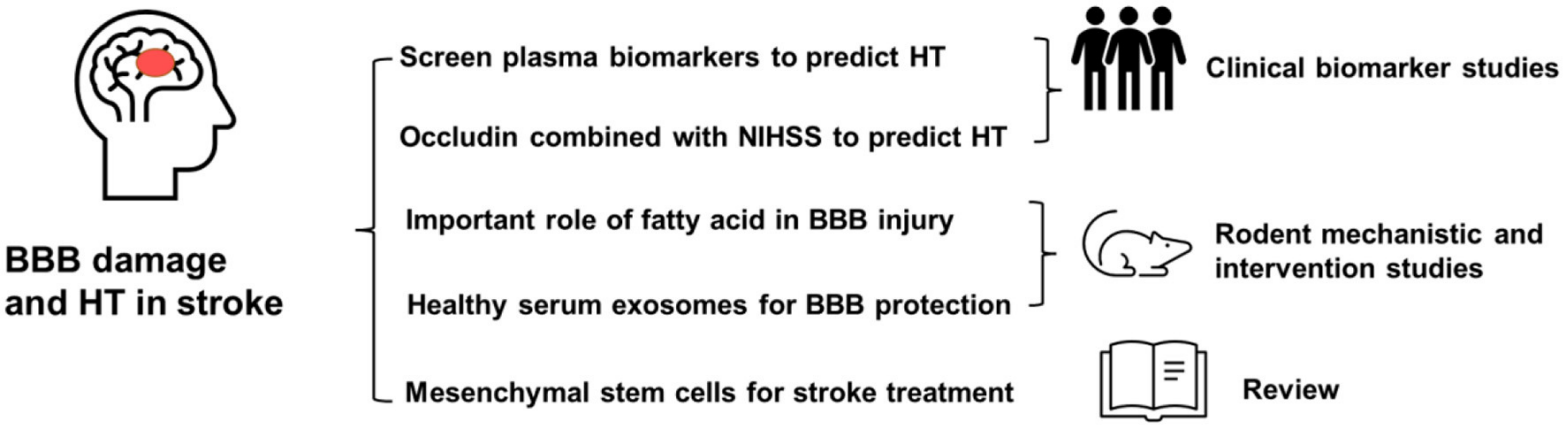
Damage to the blood-brain barrier is important in ischemic brain injury and hemorrhagic transformation. This issue focuses on how to predict HT using plasma biomarkers, recent breakthroughs in BBB damage mechanisms, healthy serum exosome treatment for rodent stroke, and a review of stem cell treatment for stroke.

## Author Contributions

All authors listed have made a substantial, direct, and intellectual contribution to the work and approved it for publication.

## Funding

HC was funded by American Heart Association Postdoctoral Fellowship (916011). YG was funded by Hainan Provincial Key Research and Development Program (ZDYF2019196) and National Natural Science Fund of China (81960227).

## Conflict of Interest

The authors declare that the research was conducted in the absence of any commercial or financial relationships that could be construed as a potential conflict of interest.

## Publisher's Note

All claims expressed in this article are solely those of the authors and do not necessarily represent those of their affiliated organizations, or those of the publisher, the editors and the reviewers. Any product that may be evaluated in this article, or claim that may be made by its manufacturer, is not guaranteed or endorsed by the publisher.
